# Genomic insights and comparative analysis of *Colletotrichum* species associated with anthracnose fruit rot and crown rot of strawberry in North Carolina

**DOI:** 10.3389/fmicb.2025.1515894

**Published:** 2025-02-10

**Authors:** Tika B. Adhikari, Norman Muzhinji, Ashley N. Philbrick, Frank J. Louws

**Affiliations:** ^1^Department of Entomology and Plant Pathology, North Carolina State University, Raleigh, NC, United States; ^2^Department of Plant Sciences, Plant Pathology Division, University of the Free State, Bloemfontein, South Africa; ^3^Department of Horticultural Science, North Carolina State University, Raleigh, NC, United States

**Keywords:** anthracnose, crown rot, *Colletotrichum*, whole genome sequencing, comparative genomics, fruit rot, strawberry

## Abstract

*Colletotrichum* is a large genus of fungal phytopathogens responsible for significant economic losses in numerous crops globally. These pathogens exhibit varying host specificities; some have a broad host range, while others are more limited. To explore the genetic composition and underlying factors of fungal virulence and pathogenicity, we sequenced the genomes of seven isolates of *Colletotrichum* spp.: three from the *C. acutatum* and four from the *C. gloeosporioides*. These isolates were sourced from anthracnose fruit rot and crown rot of strawberry in North Carolina. Phylogenetic and phylogenomic analyses classified the isolates within the *C. acutatum* as *C. nymphaeae*, while those in the *C. gloeosporioides* were identified as *C. siamense*. The genome sizes of the *C. nymphaeae* isolates ranged from 50.3 Mb to 50.7 Mb, with 14,235 to 14,260 predicted protein-coding gene models. In contrast, the genome sizes of the *C. siamense* isolates ranged from 55.7 Mb to 58.6 Mb, with predicted protein-coding gene models ranging from 17,420 to 17,729. The GC content across all genomes spanned from 51.9 to 53.7%. The predicted gene models included effectors (339 to 480), secondary metabolic gene clusters (67 to 90), and carbohydrate-active enzymes (800 to 1,060), with *C. siamense* isolates exhibiting the highest numbers in these categories. The genomic resources from this study will aid in resolving taxonomic challenges associated with *Colletotrichum* spp., elucidate their evolutionary history, and enhance the understanding of fungal biology and ecology, which is crucial for developing effective disease management strategies.

## Introduction

Strawberry (*Fragaria* × *ananassa* Duch.) is a widely grown small fruit crop, contributing significantly to economic and nutritional aspects. The strawberry industry has experienced notable growth in the United States, establishing itself as a vital element of fruit production (Samtani et al., 2019). However, this expansion faces threats from anthracnose disease caused by *Colletotrichum* spp. (Crouch et al., 2014). The *Colletotrichum* genus encompasses numerous important plant pathogens, endophytes, and saprobes (Jayawardena et al., 2016), and is categorized into species complexes comprising closely related yet distinct fungal species (Crouch et al., 2014). These species are globally distributed and found on various plants in both the tropical and temperate regions. Notably, species within the *C. gloeosporioides* (Penz.) Penz. & Sacc and *C. acutatum* J. H. Simmonds complexes are responsible for economically significant anthracnose fruit rot (AFR) and crown rot (ACR) diseases in strawberry, respectively (Ji et al., 2022). AFR is characterized by sunken black spots and necrotic lesions on petioles, stolons, and fruit, along with abundant acervuli conidia while crown rot manifests as reddish-brown necrotic areas in the crown (Brooks, 1931).

Identifying *Colletotrichum* species can be challenging due to the similarities in their morphological characteristics, which necessitate expert knowledge and experience (Khodadadi et al., 2020). Advances in molecular techniques, phylogenomic, and whole-genome sequencing have improved the classification and understanding of *Colletotrichum* as a species complex with a shared evolutionary ancestry. Multi-locus sequence analyses classify the genus into 280 species, grouped into 16 main species complexes: *C. acutatum*, *C. boninense*, *C. caudatum*, *C. dematium*, *C. agaves*, *C. destructivum*, *C. dracaenophilum*, *C. gigasporum*, *C. graminicola*, *C. gloeosporioides*, *C. magnum*, *C. orbiculare*, *C. orchidearum*, *C. spaethianum*, *C. truncatum* and *C. bambuscola* (Jayawardena et al., 2016; Cannon et al., 2012; Jayawardena et al., 2021; Chen et al., 2022; Liu et al., 2022). Additionally, 15 singletons have been reported. Within the *C. gloeosporioides* complex (Cg complex), 57 species have been described (Jayawardena et al., 2016; Weir et al., 2012; Talhinhas and Baroncelli, 2021), four of which *C. changpingense*, *C. fructicola*, *C. siamense*, and *C. theobromicola* (syn. *C. fragariae*); have been reported to cause strawberry diseases. In the *C. acutatum* complex (Ca complex), 41 species have been described, with *C. fioriniae*, *C. nymphaeae*, *C. godetiae*, *C. salicis*, and *C. simmondsii* associated with strawberry diseases. Additionally, *C. lineola* from the *C. dematium* species complex and singleton *C. nigrum* have also been reported to be associated with strawberry diseases (Jayawardena et al., 2016; Baroncelli et al., 2015; Wang et al., 2019).

Research on *Colletotrichum* spp. has mainly concentrated on their taxonomy by analyzing multigene families (Khodadadi et al., 2020). With the growing number of genome-sequenced species, numerous essential genes and virulence factors related to host infection and disease development have been identified (Baroncelli et al., 2015). Currently, the NCBI database contains 313 genomes of *Colletotrichum* spp. from various host plants. These whole-genome sequences enhance our understanding of plant-pathogen interactions and the dual lifestyles -pathogen and endophyte - exhibited by *Colletotrichum* (Gan et al., 2019). However, complete genomes have only been sequenced from a limited number of isolates from small fruits, such as *C. fiorinea* and *C. fructicola*, (Baroncelli et al., 2015; Gan et al., 2019; Armitage et al., 2020). This limited genomic representation of *Colletotrichum* from small fruit species hampers comparative genomic analyses both within and across species complexes. Despite this limitation, access to whole-genome sequences is crucial for elucidating the genomic architecture and pathogenicity gene repertoire of *Colletotrichum* spp. in strawberry, significantly enhancing our understanding of their taxonomy, virulence, and pathogenicity. Furthermore, the study of gene clusters, involved in secondary metabolism, and genome arrangements contributes to insights into fungal genome evolution and ecological interactions between *Colletotrichum* spp. and their plant hosts.

Anthracnose fruit rot (AFR) and anthracnose crown rot (ACR) are the most devastating diseases of strawberry in North Carolina, caused by *C. acutatum* and *C. gloeosporioides*, respectively (Rahman et al., 2015; Rahman et al., 2019). Multilocus sequence analysis (MLSA) classified these fungi into two species complexes: Acutatum and Gloeosporioides (Weir et al., 2012; Baroncelli et al., 2015; Damm et al., 2019). This study aimed to characterize the genome content of seven *Colletotrichum* isolates collected from diseased strawberry fields in North Carolina. These genome resources for *Colletotrichum* isolates from strawberry, which are underrepresented in databases, will facilitate comparative genomic studies on host-pathogen interactions and factors influencing host disease resistance in the host. Furthermore, the findings will aid in developing species-specific diagnostic assays, addressing challenges posed by species complexes.

## Materials and methods

### Fungal isolations

Seven isolates of *Colletotrichum* were collected from strawberry in North Carolina between 2009 and 2010: three from fruit rot (6Ca, 34Ca, and 40Ca), and four from crown rot (28Cg, 57Cg, 58Cg, and 84Cg). The procedure for isolating these fungi has been outlined in our previous studies (Adhikari et al., 2019; Jacobs et al., 2020). Furthermore, information about the fungal isolation process, the tissue of origin (such as leaf, fruit or crown), identification methods, and the locations where the isolates were collected can be found in our previous publication (Jacobs et al., 2020). Briefly, small pieces of infected fruit and crown rot from strawberry seedlings were washed in sterile water and surface-sterilized with 1% sodium hypochlorite solution to obtain pure cultures. The sterilized pieces were then placed on potato dextrose agar (PDA) and incubated at 25°C for 7 days, using a single spore method to obtain pure cultures. Morphological characteristics and pathogenicity tests were assessed (Gunnell and Gubler, 1992; Smith and Black, 1990). Identification was performed using TaqMan real-time polymerase chain reaction (TaqMan PCR) assays as described previously (Garrido et al., 2009). Some of these isolates play a significant role in strawberry breeding program (Jacobs et al., 2019; Jacobs et al., 2020). Fungal plugs were preserved at −80°C for future use.

### DNA extraction and sequencing

Mycelium was scraped from pure cultures of *Colletotrichum* isolates. Total genomic DNA was extracted using the DNeasy Plant Mini Kit (Qiagen, Germantown, MD, United States) according to the manufacturer’s recommended protocol. The constructed libraries were sequenced on a NovaSeq System (Illumina, USA), producing paired-end reads of 2 × 151 bp. The quality of the sequenced libraries was assessed using FastQC v0.11.7 (Babraham Bioinformatics, Babraham Institute, Cambridge, United Kingdom). Reads were filtered and trimmed with Fastp (Chen et al., 2018), applying a minimum Phred score of 30.

### Genome assembly and mapping

The genomes were trimmed and assembled using SPAdes v3.15.5 (Bankevich et al., 2012). Contigs shorter than 200 bp were filtered using SeqKit v0.10.1 (Shen et al., 2016). The filtered reads were then subjected to scaffolding using the Ragtag pipeline (Alonge et al., 2022), with *Colletotrichum siamense* HNO8^1^ as the reference genome. The Burrow-Wheeler Aligner (BWA -v0.7.18 and SAMtools; Li et al., 2009) were then used to sort and index the BAM files. Genome statistics were determined using QUAST v5.0.2 (Gurevich et al., 2013). To evaluate genome completeness, BUSCO analyses were performed using the fungal_odb10 and ascomycota_odb10 lineages (Manni et al., 2021).

### Phylogenetic and phylogenomic analysis

To identify the sequenced isolates, we conducted a phylogenetic analysis using five DNA barcoding regions: the 5.8S nuclear ribosomal gene along with its two flanking internal transcribed spacers (ITS), beta-tubulin (TUB2), an intron of glyceraldehyde-3-phosphate dehydrogenase (GAPDH), a partial sequence of the actin gene (ACT), and chitin synthase 1 (CHS1). We extracted the sequences of these genes from the genomes of each of the seven assemblies of *Colletotrichum* isolates. Additionally, we downloaded reference sequences for these gene regions from contemporary phylogenetic studies of *Colletotrichum* (Jayawardena et al., 2021; Liu et al., 2022; Zhang et al., 2023) from NCBI GenBank. The accession numbers for the sequences and the names of the species complexes were also obtained from NCBI GenBank (Supplementary Table S1).

We utilized OrthoVenn 3 (Sun et al., 2023) to identify orthologous protein groups for analyzing protein sequences from 28 species of *Colletotrichum*, which we downloaded from JGI Mycocosm and NCBI GenBank (Supplementary Table S2). We used *Verticillium dahliae* VdLs.17 as an outgroup (Klosterman et al., 2011). The orthologous proteins were clustered, and a species tree was generated for the *Colletotrichum* isolates. Our analysis focused on amino acid sequences corresponding to 9,137 single-copy core genes, which were extracted from the output files of OrthoVenn 3. We concatenated and aligned both trees’ DNA and protein sequences using MAFFT v.7.215 (Katoh et al., 2002), followed by manual refinement in MEGA v.11 (Tamura et al., 2021). Maximum-likelihood species phylogeny was inferred from multigene families with RA × ML v.8.2.11, including 1,000 bootstrap replicates, while MEGA v.11 was employed for analyzing multigene families. The resulting trees were visualized using iTOL version 5 (Letunic and Bork, 2021).

### Genome alignments and synteny

To assess the alignments and collinearity between genome sequences, we used D-GENIES (Cabanettes and Klopp, 2018) using Minimap2 version 5 (Li, 2018) for alignments. The MCScanX toolkit (Wang et al., 2012) was used to create chromosomal “blocks” that illustrate conserved gene orders (synteny) based on collinearity relationships. For the BlastP analysis, we used eight CPU threads, set an E-value threshold of 1 × 10^-10, and required a minimum of five Blast hits. Finally, we visualized the synteny based on the collinear blocks using SynVisio (Bandi and Gutwin, 2020).

### Identification of repetitive elements

Before gene prediction, the repetitive elements in the genome were identified using homology-based and ab initio methods. The ab initio method, RepeatModeler v.1.0.11,^2^ was used to predict repetitive sequences across all seven genomes. The predicted repetitive sequences were combined with RepeatMasker v.4.0.9,^3^ identifying repeats that matched entries in the Dfam_3.7 and RepBase-20181026 repeat library databases. Furthermore, a Tandem Repeat Finder (Maddi et al., 2022) was employed to identify repeated regions.

### Genome annotation

Gene prediction for the genomes of seven isolates of *Colletotrichum* spp. was conducted using the Funannotate v1.8.17 pipeline, following the primary assembly preparation protocol.^4^ This process involved cleaning repetitive contigs, sorting the assembly by length, applying repeat masking, and gene prediction. Gene models were generated using AUGUSTUS, an ab initio gene predictor, alongside all fungal proteins available in the SwissProt database, which were manually curated as of March 18, 2024. These proteins were further categorized using InterProScan v5.66–98.0 (Jones et al., 2014), and KEGG (Kyoto Encyclopedia of Genes and Genome; Kanehisa et al., 2016). The ribosomal RNA subunits were analyzed with RNAmmer v1.2 (Lagesen et al., 2007), and tRNA genes were identified utilizing tRNAscan-SE v14 with the RNAweasel interface (Lowe and Eddy, 1997).^5^

Predictions of genes encoding potential secreted proteins were conducted using a combination of SignalP v6, TargetP v1.1, and TMHMM v2.0, integrated into a deep neural network-based software SECRETOOL.^6^ The secretome was defined as the aggregated results of SignalP and TargetP, filtered based on TMHMM to include proteins with zero or one transmembrane domain. Additionally, EffectorP v3.0 (Sperschneider and Dodds, 2022) was used to identify potential effector proteins within the predicted secretome. Effector candidate were classified as predicted secreted proteins containing a signal peptide but lacking transmembrane domains or glycosylphosphatidylinositol anchors, and limited to a maximum of 300 amino acids. BLASTP analysis was performed against the Swiss-Prot database with a cutoff E-value of 10^-5 to assess the similarity of effector candidates to known proteins.

To identify gene clusters associated with secondary metabolite production, we employed the antiSMASH (Antibiotics and Secondary Metabolite Analysis) tool (Blin et al., 2023), focusing exclusively oncore biosynthetic genes (SMKGs). Carbohydrate-active enzymes (CAZymes) annotated using the dbCAN2 server (Zheng et al., 2023), which utilized HMMER, DIAMOND, and eCAMI (Yin et al., 2012). Genes were designated as CAZymes when at least two of the three tools provided a positive annotation. To reduce false positives, the identified CAZymes were analyzed using InterProScan 5 with an E-value cutoff of 1e-10, and the member counts for each CAZyme family were adjusted accordingly. Furthermore, the predicted proteins were compared with the Pathogen-Host Interaction (PHI) database, Version 4.7^7^ (May 2024), using BLASTP with an E-value cutoff of 1e-5.

## Results

The genome sizes of seven *Colletotrichum* spp. isolates ranged from 50.3 Mb for isolate 40Ca to 58.6 Mb for isolate 58Cg (Table 1). The *C. siamense* isolates had larger genome, ranging from 56.1 to 58.6 Mb, while the *C. nymphaeae* isolates had smaller genomes ranging from 50.3 to 50.7 Mb. The contig N50 lengths varied from 167,900 kb to 202,057 kb, and the largest contig sizes ranged from 668,652 kb for isolate 40Ca to 667,855 kb for isolate 28Ca (Table 1). The GC content of the sequenced genomes was between 51.9 and 53.7%, with isolates 6Ca and 84Cg representing the lower and upper extremes, respectively. All genomes exhibited high completeness levels, ranging from 98.2 to 99.1% (Table 1). Isolates of *C. nymphaeae* from the *C. acutatum* showed smaller genome assemblies compared to those of *C. siamense* from the *C. gloeosporioides*. The annotated genome sequences of the *Colletotrichum* isolates have been deposited in GenBank under BioProject PRJNA1090952 and BioSample accession numbers SAMN40582474 and SAMN40582476-8 (Table 1).

**Table 1:** Summary of the genome statistics for the seven *Colletotrichum* isolates obtained from strawberry in North Carolina.

		*C. nymphaeae*			*C. siamense*			
	Feature	6Ca	34Ca	40Ca	28Cg	57Cg	58Cg	84Cg
Genome annotation	Genome size (Mb)^a^	50.9	51	50.3	56.1	57.8	58.6	56.5
	Genome coverage	87×	103×	58×	75×	68.0×	98×	80×
	GC (%)^b^	51.98	51.98	52.60	53.50	53.50	52.90	53.70
	# Contigs	958	1,002	958	2,391	2,270	973	1,002
	Largest contigs (kb)	668,652	667,855	668,652	623,307	572,964	816,516	762,990
	N50^c^ contig length (kb)	183,114	183,989	183,114	174,300	167,900	202,057	197,661
	L50^d^	88	88	83	93	100	92	90
	N90	47,203	47,203	52,516	51,290	46,765	46,742	51,178
	L90	288	288	265	297	330	331	332
	Repetitive elements (%)	1.12	1.22	1.15	1.25	1.14	1.27	1.15
Genome completeness	Busco genome completeness (%) ^e^	99.1	99.1	98.2	99.7	99.4	98.9	99.2
	BioSample Accession number	SAMN40582474	SAMN40582476	SAMN4058247	SAMN40582478	SAMN40582479	SAMN40582480	SAMN40582481
	SRA accession numbers	SRR29996184	SRR29996182	SRR29996181	SRR29996180	SRR29996179	SRR29996178	SRR29996182
	GenBank accession #	-^f^	-	-	GCA_040955545.1	GCA_040955525.1	-	GCA_040955925.1

^a^Genome size is calculated from the total sequence length of the final processed reads.^b^GC% calculated from the final assembly of processed reads.^c^N50 is defined as the sequence length of the shortest scaffold at 50% of the total assembly length.^d^L50 is defined as the number of scaffolds whose summed length equals 50% of the total assembly length.^e^Benchmarking Universal Single-Copy Orthologs (BUSCO).^f^GenBank accession numbers not available.

**Figure 1:**
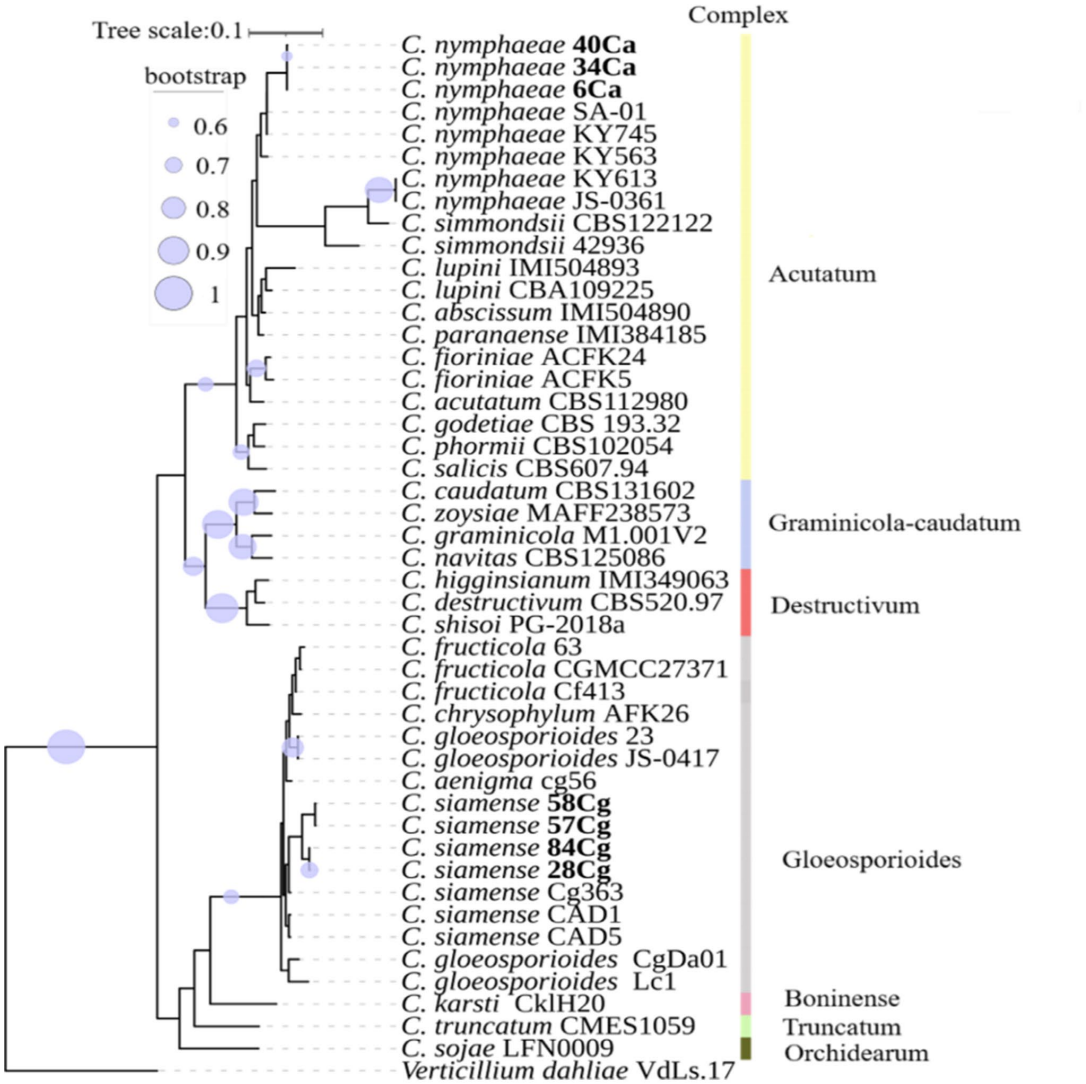
A phylogenomic tree constructed using single-copy orthologous genes illustrates the relationship of *Colletotrichum* spp. with additional *Colletotrichum* isolates from NCBI (Supplementary Table S2). The nodes in the tree depict maximum likelihood (ML) bootstrap support values ≥60% from 1,000 replicates. The isolates studied are highlighted in bold. Different color boxes indicate isolates belonging to various *Colletotrichum* spp. complexes.

Phylogenetic and phylogenomic analyses, using the combined data set of five gene regions, revealed that isolates in the Acutatum complex grouped and clustered with *C. nymphaeae* with a bootstrap support of more than 95% (Supplementary Figure S1). All four isolates in the Gloeosporioides complex formed a sister relationship with *C. siamense* with bootstrap support of more than 96% (Figure 1; Supplementary Figure S1). It is worth noting that the tree topologies of both the multigene family and genomics are identical.

The proportion of repetitive elements in the sequenced *Colletotrichum* genomes did not vary significantly. The repeat content of the isolates ranged from 1.12% in *C. nymphaeae* 6Ca to 1.27% in *C. siamense* 84Ca, with interspersed repeats constituting 0.06% of the total genome (Table 1; Supplementary Table S3). Gene prediction analysis revealed that the average gene count for *C. nymphaeae* isolates within the *C. acutatum* ranged from 14,152 to 14,260. Isolates belonging to the *C. gloeosporioides* showed similar trends. In contrast, *C. siamense* isolates within the *C. gloeosporioides* had more predicted gene models, ranging from 17,420 to 17,729 (Table 2).

**Table 2:** Genome assembly and functional annotation statistics of the seven *Colletotrichum* isolates from strawberry in North Carolina.

		*C. nymphaeae*			*C. siamense*			
	Feature	6Ca	34Ca	40Ca	28Cg	57Cg	58Cg	84Cg
Gene prediction	Predicted protein models	14,235	14,152	14,260	17,420	17,686	17,673	17,729
	Coding sequences	13,901	13,801	13,905	16,922	17,196	17,280	17,318
	tRNA	334	351	355	498	490	393	411
Gene annotation	rRNA	24	24	24	28	33	34	29
	Secretome	1,118	1,112	1,121	1,336	1,351	1,354	1,353
	Predicted CAZymes	800	1,002	958	1,060	1,043	973	1,002
	Predicted effectors	347	352	339	471	478	480	479
	Secondary metabolites gene clusters	67	72	71	90	87	88	91

### Comparative genome analysis and sequence synteny

**Figure 2:**
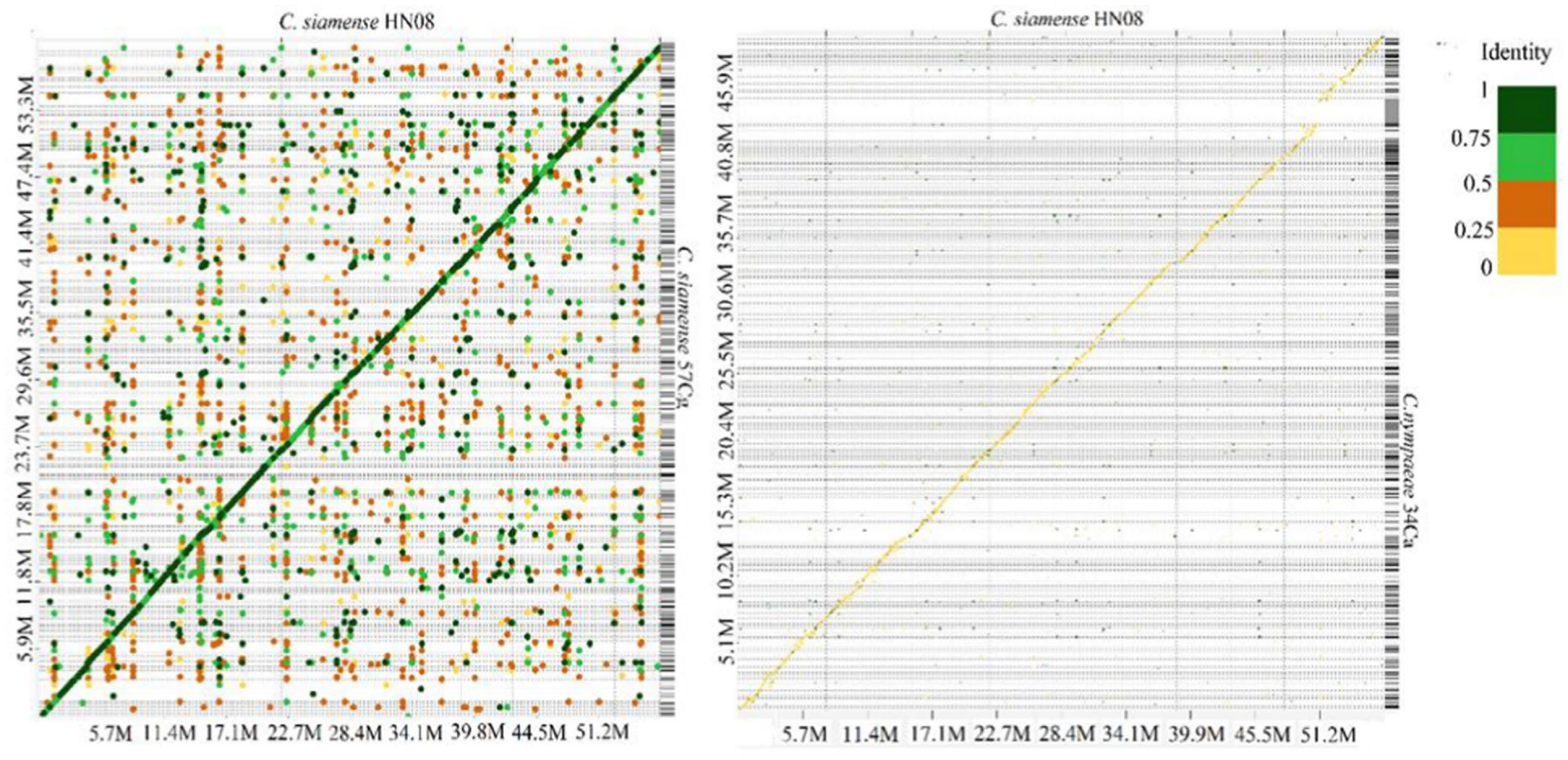
A Dotplot image comparing the *C. siamense* 57Cg **(A)** *C. nymphaeae* 34Ca **(B)** and with *C. siamense* HN08 reference chromosomes. Dotplot analysis was conducted using the minimap2 aligner, and the result was subsequently visualized on the D-GENIES platform (https://dgenies.toulouse.inra.fr). The colors in the dot plot represent identity levels.

*Colletotrichum* genomic sequences were analyzed using MCScanX (Wang et al., 2012) and D-Genies (Cabanettes and Klopp, 2018). In the D-Genies plot (Figure 2A) the diagonal line indicates regions of high sequence identity, suggesting conserved sequences between the two genomes However, *C. siamense* HN08 and *C. nymphaeae* 34Ca exhibit low sequence similarity with deviations from diagonal line, indicating frequent insertions or deletions (Figure 2B). The genome synteny and collinearity analysis of isolates 40Ca, and 57Cg isolates shows 73.18% shared collinear genes (Supplementary Figure S2).

**Figure 3:**
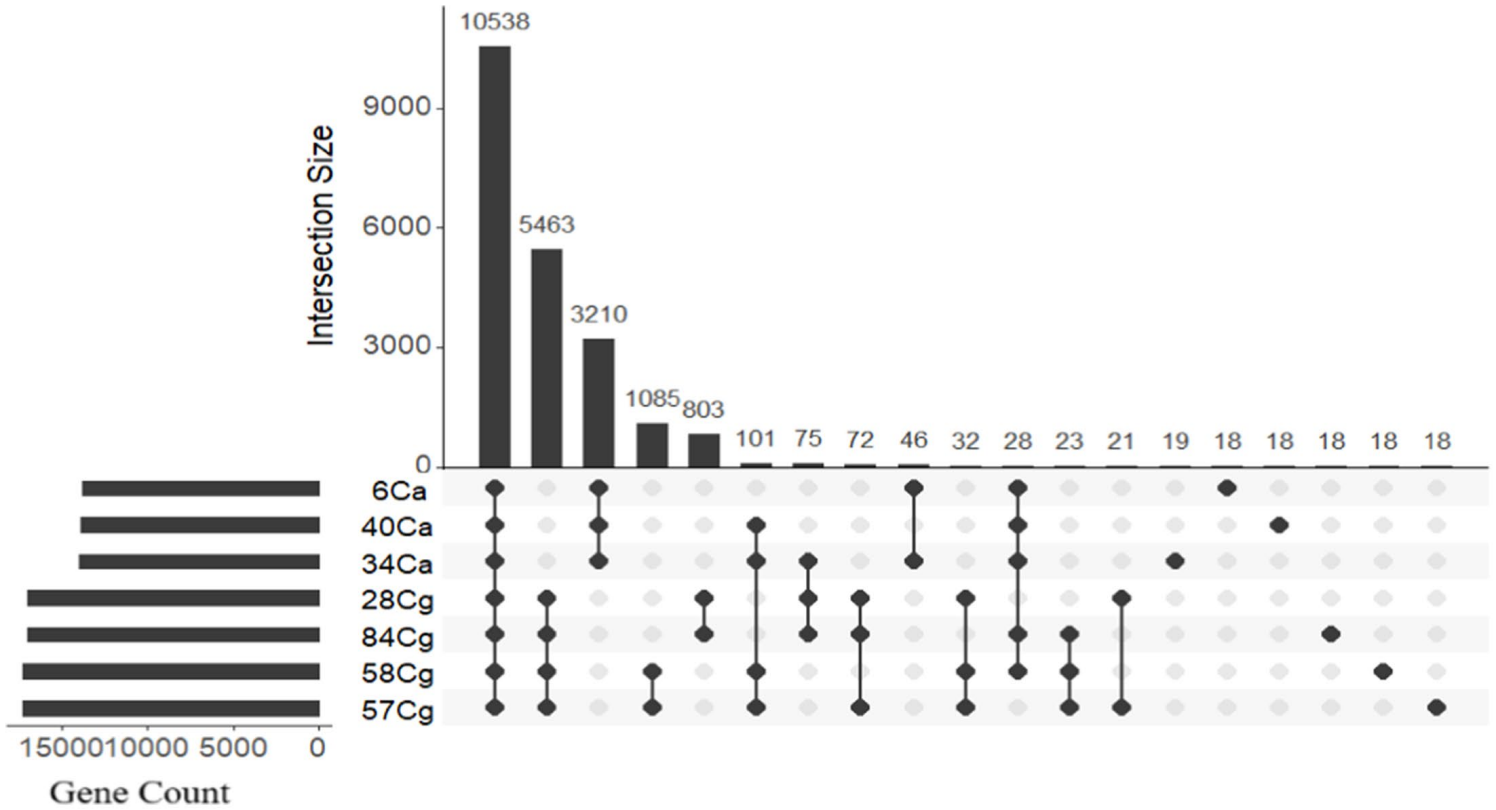
The distribution of genes in *Colletotrichum* orthologs reveals a frequency pattern that highlights genes conserved in only a few genomes, along with orthology groups that consist of a single gene per isolate. OrthoVenn 3 was used to categorize the genes into ortholog clusters, which are illustrated in the bar graphs displaying all ortholog groups. An UpSet table shows both unique and shared orthologous clusters among the isolates. The horizontal bar chart represents the number of orthologous clusters per isolate, while the vertical bar chart on the right indicates the number of clusters shared among the isolates. Lines in the chart illustrate intersecting sets.

The protein functions of the seven *Colletotrichum* isolates were evaluated by clustering proteins into homologous families known as orthogroups. Analysis of the protein-coding genes showed that the *Colletotrichum* genomes contained 20,103 orthologous gene families, comprising a total of 112,951 genes. This represented 99.7% of all predicted genes (113,284) (Figure 3; Supplementary Table S4). Among these, 10,278 gene families were shared by all seven isolates and were categorized as core gene families. Orthofinder identified proteins organized into 9,137 single orthogroups, with eight orthogroups being species-specific and encoding 198 genes (Figure 3; Supplementary Table S4).

**Figure 4:**
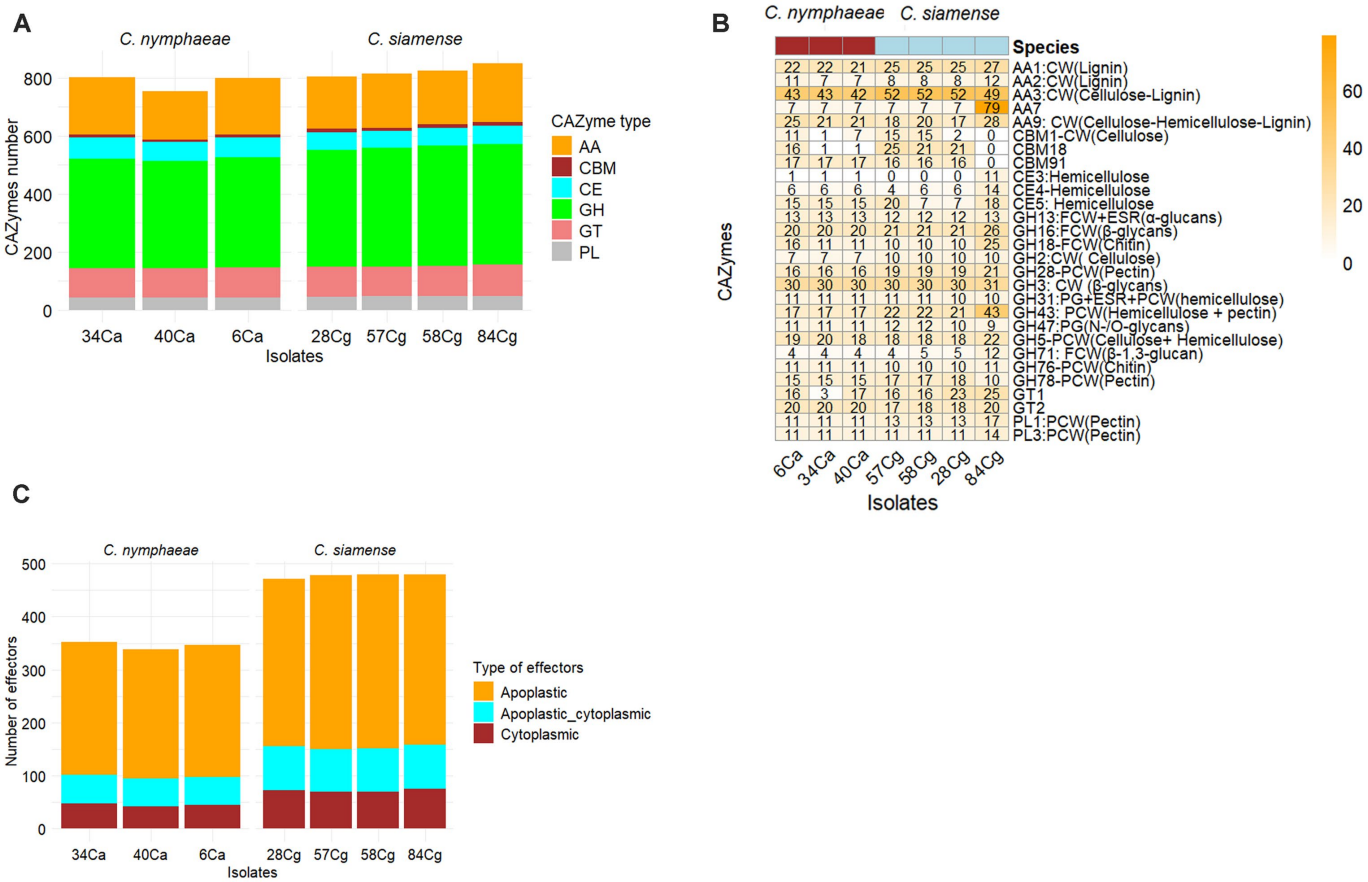
The predicted number of CAZymes (Carbohydrate Active Enzyme) in each *Colletotrichum* isolate. CAZyme families shown are AA-auxiliary activities, CB-carbohydrate-binding, CE-carbohydrate esterases, GH-Glycoside hydrolases, GT-glycosyltransferases, PL-polysaccharide lyases, and CBMs - carbohydrate-binding modules **(A).** Heatmap showing the comparative analysis frequency of seven isolates of *Colletotrichum* CAZyme families in their genomes. Only CAzymes with a frequency greater than 10 are shown. Hierarchical clustering was performed with Euclidean distance and Ward linkage. Overrepresented and underrepresented CAZyme families are indicated in red, orange, and blue, respectively **(B).** Predicted number of effectors in each *Colletotrichum* isolate **(C).**

This study identified and analyzed the secreted proteins, CAZ enzymes, effectors, secondary metabolite synthesis gene clusters, and effectors in the genomes of each strain. The number of secreted proteins ranged from 1,112 to 1,121 for *C. nymphaeae* isolates, while the secretome gene count ranged from 1,336 to 1,354 for *C. siamense* isolates (Table 2). The CAZymes of three isolates belonging to the *C. nymphaeae* ranged from 800 to 1,002, whereas the numbers of these potentially pathogenic genes were significantly higher in *C. siamense* isolates, ranging from 973 to 1,060 CAZymes (Table 2; Figure 4A; Supplementary Table S5). Isolate 28Cg had the highest number, while 40Ca had the lowest number. CAZymes play an essential role in facilitating the acquisition of complex carbohydrate metabolism. Most of the genes identified encode glycoside hydrolases (GHs) (41%), glycosyltransferases (GTs) (16%), auxiliary activity (AAs) (16%), carbohydrate-binding modules (CBMs) (12%), carbohydrate esterases (CEs) 8% and polysaccharide lyases (PLs) 3% (Figure 4A; Supplementary Table S5).

Isolates *C. nymphaeae* 6Ca and *C. siamense* 84Cg have the lowest and highest distribution, respectively. In these families, enzymes that break down lignocellulose and cellulose-like GHs 1, 2, 3, 7, 9, 10, 12, 74, and AA9 were identified. Lignin-modifying enzymes (LMEs) were mainly distributed in families AA1 and AA2. Across all seven genome assemblies, the CAZyme family’s AA3 and the GH3 of gluco-oligosaccharide oxidases were the most abundant. CAZyme AA3 is typically found in wood-degrading fungi (Ludwig and Kracher, 2024), whereas the GH3 family is associated with breaking down and debranching hemicellulose, such as glucosidase, xylosidase, and glucanase activities. This family encompasses a variety of functions, including members with *β*-D-glucosidase, β-D-xylopyranosidase, *α*-L-arabinofuranosidase, and N-acetyl-β-D-glucosaminide phosphorylase activities, which are essential in degrading oligosaccharides. The Auxiliary Activity CAZyme Families AA1, AA2, AA9, AA3, and AA7 were much more frequent in the 84Cg assembly (Figure 4B). This same trend was observed in the Glycoside hydrolase families, with GH16, GH43, and GH71 showing significantly higher frequencies in 85Cg (Figure 4B).

Effectors predicted via EffectorP-Fungi v.3.0 were classified as apoplastic, cytoplasmic, and dual-predicted apoplastic/cytoplasmic effectors (Figure 4C). More than 70% of the effectors were classified as apoplastic. Consistent numbers of each effector type were detected in each isolate from the same complex. Generally, isolates belonging to *C. siamense* had more effectors (471 to 480) than *C. nymphaeae* isolates (339 to 347). *C. nymphaeae* isolates shared the same effectors and had no isolate-specific effectors (Table 3). However, 28Cg and 57Cg had isolate-specific effectors, such as the EC51a and Celp0028 effector-like proteins. Isolate 84Cg had seven isolate-specific effectors, five of which were uncharacterized proteins. The other two were annotated as the secreted virulence factor MC69 and the GPI-anchored serine–threonine-rich protein. The *C. nymphaeae* isolates had more NLP (3) domain effectors and fewer LysM domain proteins (33 to 34), compared to the *C. siamense*, which had 1–2 NLP domain and 56–62 LysM domain proteins (Table 3).

**Table 3:** Effectors that are shared between isolates used in this study.

Isolate	Number of effectors	Shared effectors	Isolate specific effectors	BLAST identity	Effectors with NLP domain (PF05630 and IPR008701)	Effectors with LySM motifs
6Ca	347	347	0	–	3	33
34Ca	347	347	0	–	3	34
40Ca	339	339	0	–	3	33
28Cg	471	470	1	EC51a protein	2	58
57Cg	478	477	1	Celp0028 effector-like protein	1	62
58Cg	481	480	1	Uncharacterized proteins 1 Secreted virulence factor MC69	1	59
84Cg	479	472	7	Uncharacterized proteins 1	2	56

**Figure 5:**
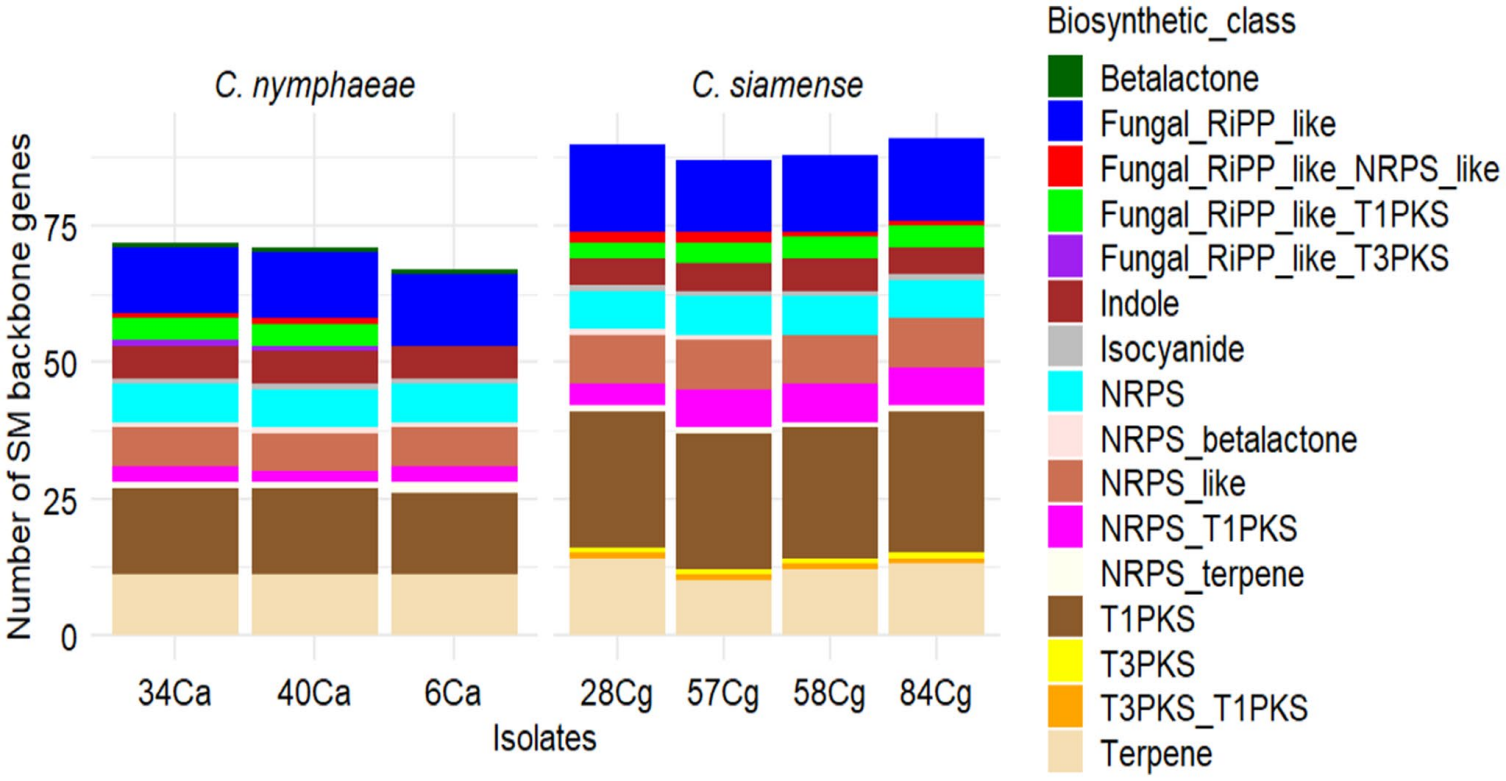
Summary of predicted secondary metabolites (SM) essential genes and clusters in seven *Colletotrichum* isolates, three *C. nymphaeae;* 6Ca, 34Ca, 40Ca belonging to the *C. acutatum* and four *C. siamense* 28Cg, 58Cg, 58Cg and 84Cg belonging to the *C. gloeosporioides*. Type I PKS (T1PKS), non-ribosomal peptide synthetase cluster (NRPS), fungal ribosomal synthesized and post-translationally modified peptide product cluster (RiPP-like), and Type 3 PKS (T3PKS).

To identify potential genes involved in the biosynthesis of secondary metabolites in the *Colletotrichum* genomes, we utilized antiSMASH v7.0 to annotate and analyze the gene clusters associated with secondary metabolite biosynthesis (BGCs). The BGC clusters ranged from 69 to 71 for *C. nymphaeae* isolates and 87 to 91 for the *C. siamense* isolates (Figure 5). The gene clusters were categorized into 17 BGC types (Figure 5; Supplementary Table S6). Among these, terpenes, Type I polyketide synthase (T1PKS), non-ribosomal peptide synthetase cluster (NRPS), and fungal ribosomal synthesized and post-translationally modified peptide product cluster (RiPP-like) were the most prevalent BGCs in the *Colletotrichum* genus. NRPS-like fragments, isocyanides, indoles, beta lactones, T3PKS, and hybrids of NRPS (terpene, T1PKS, T3PKS, and fungal-RiPP-like) were also observed, though at lower frequencies. The BGC types T3PKS and its hybrids were specific to C. *nymphaeae*, whereas beta-lactam was absent from the *C. siamense* (Supplementary Table S6).

Secondary metabolite clusters microperfuramone, nectriapyrone, alternaryrone, and xenolozoyerone had 100% similarity with known biosynthetic gene clusters, while polypyrone with 80% similarity was unique to C. *nymphaeae* (Supplementary Table S6). UNII-Y-C20Q1094PT, fusarin, chrysogen, betaenone, clavaric acid, ACT-toxin III, ACE, ipicidin, and bataestatic were unique to *C. siamense* (Supplementary Table S6). Shared secondary metabolite clusters between the two complexes include choline, cichorine, biotin, cercosporin, Sequalistin 1, and ACE (Supplementary Table S6).

The genome-wide annotations of the *Colletotrichum* isolates were analyzed using the Pathogen-Host Interaction Database (PHI). This analysis identified genes associated with reduced virulence, loss of pathogenicity, increased virulence, effector functions, and annotated chemical targets (Table 4). The *C. siamense* isolates contained more lethal genes and genes linked to reduced virulence compared to *C. nymphaeae* isolates.

**Table 4:** Pathogen Host Interaction database annotation result statistics of *Colletotrichum* isolates.

Phenotypic category	*C. nymphaeae*			*C. siamense*			
	6Ca	40Ca	34Ca	28Cg	57Cg	58Cg	84Cg
Reduced virulence	4,513	4,413	4,421	4,341	3,789	4,210	3,641
Unaffected pathogenicity	2,649	2,565	2,794	3,406	2,700	2,589	2,438
Planta virulence determinant	730	733	733	777	728	657	698
Loss of pathogenicity	341	331	333	373	372	372	373
Increased virulence	395	375	379	430	381	370	370
Lethal	146	150	147	172	172	172	172
Loss of pathogenicity	68	69	70	70	75	78	78
Chemistry target: resistance to chemical	40	28	28	32	31	29	32

**Figure 6:**
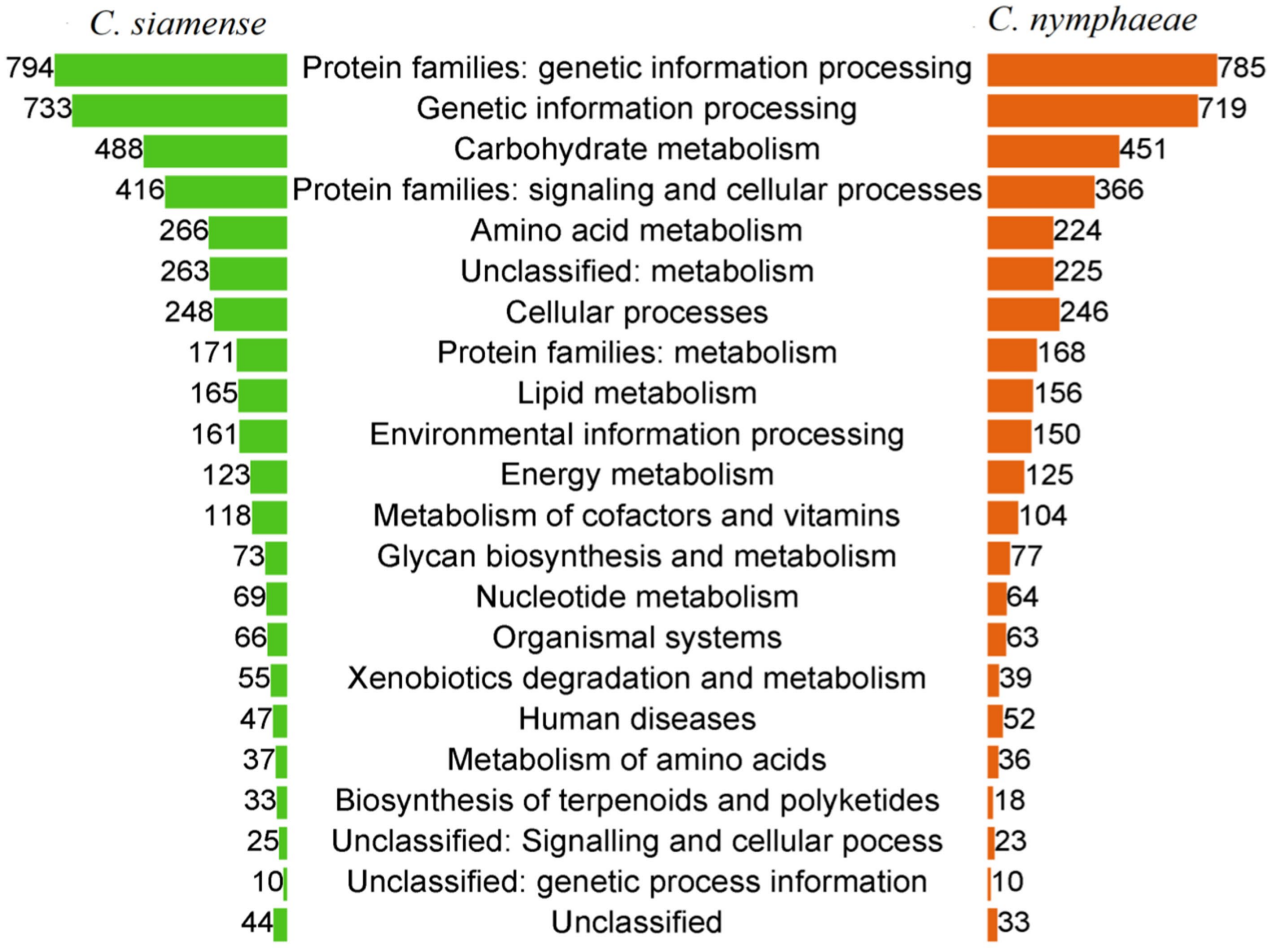
Functional annotation of the *Colletotrichum* isolates genome using the Kyoto Encyclopedia of Genes and Genomes (KEGG) database, accounting for 29.4% of the total predicted genes for the *C. siamense isolates* and 24.9% of the total predicted genes from the *C. nymphaeae.*

In the KEGG database, 4,429 genes (29.4% of the total predicted genes) were annotated for *C. siamense* isolates, and 4,162 genes (24.9%) were annotated for *C. nymphae* isolates. Most genes were related to genetic information processing (785–794), carbohydrate metabolism (451–488), cellular processes (246–248), and environmental information processing, metabolism, and organismal systems (Figure 6).

## Discussion

Strawberry anthracnose, caused by *Colletotrichum* spp., is a significant disease affecting strawberry in the United States and other countries. A detailed analysis of the genome sequence was performed to gain insights into the genomes of anthracnose-causing pathogens of strawberry in North Carolina. This study focussed on the genome profiles of *C. nymphaeae* and *C. siamense* isolates from the *Colletotrichum* complexes, Acutatum, and Gloeosporioides, respectively. Due to the limited availability of whole genome sequence information for *Colletotrichum* isolates from strawberry, our understanding of secretory proteins, including degrading enzymes, remains limited. Therefore, whole genome sequencing and annotation of the *Colletotrichum*-strawberry pathosystem are vital for advancing our understanding of this disease.

In this study, the genomes of seven *Colletotrichum* isolates were sequenced, assembled, annotated, and analyzed, with a focus on evidence of carbohydrate-active enzymes (CAZymes), effectors, and secondary metabolic clusters. The *C. siamense* isolates exhibited the largest genome sizes, ranging from 55.7 Mb to 58.6 Mb, while the *C. nymphaeae* isolates had genome sizes between 50.3 Mb and 50.7 Mb. These findings align with previous reports on other *Colletotrichum* spp. (Armitage et al., 2020; Becerra et al., 2023; Wang et al., 2023). High N50 values and BUSCO completeness metrics indicate the quality of the genome assembly. The proportion of repetitive sequences in the genomes varied from 1.12% (isolate 6Ca) to 1.27% (isolate 58Cg), compared to 1.03% reported in other *Colletotrichum* species (Lv et al., 2024). This variation was not dependent on genome size and was significantly lower than the proportions reported in other studies, 3.63% for *C. fructicola* (Armitage et al., 2020). This reduced proportion can be attributed to our sequencing and assembly strategy, which excluded scaffolds shorter than 200 bp. This study provides high-quality gene predictions and annotations, establishing a robust foundation for future genetic, population genomic, and evolutionary research.

To delineate the boundaries of the *Colletotrichum* species complex, the phylogenomic tree exhibited a topology consistent with that of the five-locus gene tree. Most species complexes formed well-supported clades, corroborating findings from previous studies (Crouch et al., 2014; Jayawardena et al., 2016). The three isolates from the *C. acutatum* formed a robust clade with *C. nymphaeae*. In contrast, isolates from the *C. gloeosporioides* clustered with *C. siamense*. These taxa are prevalent pathogens affecting various plants (Weir et al., 2012; De Silva et al., 2017) and have been documented as infecting strawberry globally (Jayawardena et al., 2016; Baroncelli et al., 2015; Wang et al., 2019; Armitage et al., 2020).

The predicted gene counts for *Colletotrichum* spp. ranged from 14,250 to 17,686, with *C. siamense* isolates displaying the highest predicted gene models. Significant genetic divergences exist between *C. nymphaeae* and *C. siamense* isolates, influencing their lifestyles and pathogenicity (Baroncelli et al., 2015). The predicted gene count for *C. siamense* exceeds that reported by Rehner et al. (2023) but is lower than the 18,324 protein-coding genes identified in *C. lupini* (Baroncelli et al., 2021). Secretome predictions for *C. nymphaeae* isolates ranged from 1,112 to 1,121, while those for *C. siamense* isolates ranged from 1,336 to 1,354. Isolates within the same complex tend to exhibit similar secretome compositions. Previous research indicates that secretome size correlates with phylogenetic relationships (Krijger et al., 2014), and variations in secretomes are associated with fungal lifestyles (Alfaro et al., 2014). Furthermore, isolates within the same species complex that possess similar genome sizes tend to have analogous secretome components. The secretome encompasses fungal virulence factors, including carbohydrate-active enzymes (CAZymes), effector proteins, secondary metabolite gene clusters, and additional effector proteins (Tsushima and Shirasu, 2022).

CAZymes are critical enzymes that facilitate the synthesis and degradation of complex carbohydrates within cells, playing a vital role in fungal biological activity and pathogenicity. The predicted CAZyme counts in *Colletotrichum* isolates ranged from 800 (6Ca) to 1,060 (28Cg), aligning with similar figures observed in other *Colletotrichum* pathogens (Armitage et al., 2020; Zhang et al., 2023). Notably, the *C. siamense* species complex exhibited the highest average CAZyme count. This abundance may elucidate the capability of species within this complex to infect 283 host plants at various developmental stages, functioning either as pathogens or endophytes (Talhinhas and Baroncelli, 2021). This observation aligns with earlier findings (Baroncelli et al., 2016) indicating that host range diversity is linked to the expansion and contraction of gene families in *Colletotrichum*.

This study identified a diverse array of glycoside hydrolases (GHs) critical for degrading glycosidic bonds in plant cell wall polysaccharides, facilitating cell wall degradation and subsequent infection. Both *Colletotrichum* complexes exhibit comparable enzyme profiles for cellulose degradation, including GH5, GH6, GH7, GH12, GH45, and GH61, and for hemicellulose breakdown, encompassing CE1, GH10, GH11, GH26, GH29, GH43, GH51, GH53, GH54, GH62, GH67, GH74, and GH93. Additionally, enzymes from the CE10, CBM1, AA9, AA3, AA7, GH5, GH16, GH28, GH43, and PL1 families were detected, underscoring the AA family’s role in plant cell wall degradation. Notably, isolate 84Cg demonstrates a high abundance of AA7 enzymes, potentially involved in the conversion or detoxification of lignocellulosic compounds. Enzymes from the AA9 and GH16 families exhibit cellulase activity, while GH28, GH43, and PL1 show pectinase activity. All fungal genomes analyzed encode comparable quantities of glycosyltransferases (GTs) necessary for fungal cellular functions, including cell wall synthesis and carbohydrate metabolism. These observations corroborate findings by O’Connell et al. (2012). The presence of lignin-degrading AA2 family enzymes suggests a role in xylem vessel infection, aiding the translocation of propagules and enhancing secondary infections. The CAZyme profiles of *C. nymphaeae* and *C. siamense* isolates resemble those of hemibiotrophic and necrotrophic plant pathogens, further emphasizing the importance of glycoside hydrolases in the infection processes of *Colletotrichum* spp (Tsushima and Shirasu, 2022).

*Colletotrichum* spp. exhibit a broad host range and utilize two primary classes of secreted effectors for infection. Apoplastic effectors, located outside plant cells, comprise glycoside hydrolases and necrosis-inducing proteins (NLPs), which are essential for compromising the plant’s physical and chemical defenses and providing necessary nutrients during early infection stages (Dong et al., 2012). In contrast, cytoplasmic effectors manipulate host physiology and morphology to increase susceptibility, including suppressing host immunity and promoting programmed cell death. These effectors typically enter plant cells via specialized structures known as haustoria. Genomic analyses reveal that 75% of the effectors are apoplastic, 10% are cytoplasmic, and 15% exhibit characteristics of both. Most effectors are conserved across isolates; however, isolate 28Cg contains a unique effector with 95% identity to a putative Celp0028 effector-like protein, contributing to the virulence and stress tolerance of *Sclerotinia sclerotiorum* (Wei et al., 2022). Isolate 84Cg possesses seven isolate-specific effectors, none shared with other isolates. Among these, five are uncharacterized proteins, while two resemble a GPI-anchored cell wall protein and the secreted protein MC69, as determined by BLAST and InterProScan analyses. The GPI-anchored cell wall protein is critical for maintaining cell wall integrity and serves as a pathogenicity determinant in many fungi. Additionally, the secreted protein MC69 is vital for the infection of both dicots and monocots and is essential for the full virulence of *C. orbiculare* (Irieda et al., 2016).

A BLASTP analysis utilizing the Swiss-Prot database identified several effector candidates containing necrosis-inducing proteins (NLPs) and lysin motifs (LysMs), as determined by the LysM domains identified through InterProScan (Jones et al., 2014). All isolates exhibited effector proteins with NLP activity, identified through searches using IPR (InterPro) and PFAM terms while cataloging domains with supplementary IPR and PFAM annotations. Isolates of *C. nymphaeae* contained three NLP proteins, while *C. siamense* exhibited one. NLPs are known to induce leaf necrosis and activate immune responses in dicotyledonous plants (Pemberton and Salmond, 2004). Furthermore, all isolates possessed LysM-encoding genes, with counts ranging from 33 to 34 in *C. nymphaeae* and from 56 to 62 in *C. siamense*. LysMs interact with chitin and peptidoglycans, sequestering chitin fragments released during fungal infections in the apoplastic space, thereby inhibiting the detection of these fragments by plant immune receptors (Krijger et al., 2014).

Fungi synthesize various secondary metabolites (SMs) crucial for environmental interactions and organismal communication (Keller et al., 2005). These metabolites encompass polyketides (PKs), non-ribosomal peptides (NRPS), terpenes (TS), isocyanates, indoles, beta-lactones, and fungi-RiPP. They serve as protective agents against stress and significantly influence pathogenicity and virulence. This study identified six prominent groups of secondary metabolites produced by *Colletotrichum* isolates: PKs, NRPS, TS, non-canonical isocyanide synthase, trimodular NRPSs, and fungi-RiPP. Furthermore, PKS, NRPS, and PKS-NRPS hybrids were detected among the isolates. Most secondary metabolite clusters in the sequenced isolates were reminiscent of type I PKs, typically associated with fungal toxin biosynthesis (Keller et al., 2005). The predominant secondary metabolite gene clusters (SMGCs) observed were 15 in *C. nymphaeae* and 25 in *C. siamense*. Unique SM clusters in *C. nymphaeae* included microperfuramone, nectriapyrone, alternaryrone, and xenolozoyerone. Notably, alternaryrone, related to alternariol, exhibits mycotoxin properties and induces oxidative stress in plant cells, thus enhancing the pathogenicity of *C. nymphaeae* (Talhinhas and Baroncelli, 2021).

*Colletotrichum siamense* isolates produce distinctive secondary metabolites, including UNII-Y-C20Q1094PT, fusarin, chrysogen, betaenone, clavaric acid, and ACT-toxin III. Betaenone exhibits phytotoxic effects, such as chlorosis and necrosis in host plants. Clavaric acid inhibits squalene synthase, disrupting sterol biosynthesis and compromising the host plant’s cellular integrity, which facilitates fungal invasion (Baroncelli et al., 2016). ACT-toxin III comprises host-specific toxins that target susceptible plant varieties, resulting in cell death and tissue necrosis, thereby allowing *efficient* infection of specific plant hosts by *C. siamense* (Dowling et al., 2020).

Both species within the *Colletotrichum* complex possess specific clusters of secondary metabolites (SM), including choline, chlorine, biotin, cercosporin, Sequalistin 1, and ACE, which are critical for the survival and virulence of pathogenic fungi (Krijger et al., 2014). The choline and scytalone/T3HN biosynthetic gene clusters reside on chromosome 2. Choline is essential for phospholipid formation, and vital for cell membranes. In *Colletotrichum*, choline may aid in maintaining cellular integrity during infection. Cichorine, a phytotoxic compound, inhibits plant growth and defense responses (Keller et al., 2005). A cercosporin-like biosynthetic gene cluster has also been mapped to chromosome 7. Cercosporin generates reactive oxygen species (ROS) that damage host plant cells, further facilitating infection. Sequalistin 1 and ACE inhibit proteases, enabling *Colletotrichum* to circumvent plant defense mechanisms. Collectively, these secondary metabolite profiles bolster *Colletotrichum’s* hemibiotrophic and necrotrophic lifestyle (Talhinhas and Baroncelli, 2021).

## Conclusion

The seven sequenced genomes of *Colletotrichum* isolates augment the genome database and facilitate comparative genomics for investigating host specificity, pathogenicity mechanisms, and evolutionary history. They also contribute to the development of diagnostic tools and population genetic markers. These resources will bolster future research on strawberry - *Colletotrichum* interactions, providing a foundation for studies on effector biology, secondary metabolites, transposable elements, and evolutionary population genomics.

## Data Availability

The authors state that all the necessary data for confirming the conclusions presented in the article are presented fully within the article and supplemental material. The raw sequencing data for the genome and the assembly reported in the paper is associated with BioProject number PRJNA1090952 and BioSample numbers SAMN40582474, SAMN40582476, SAMN4058247, SAMN40582478, SAMN40582479, SAMN40582480, and SAMN40582481.

## Footnotes

^1^ https://www.ncbi.nlm.nih.gov/datasets/genome/GCA_038023885.1/

^2^ http://www.repeatmasker.org

^3^ www.repeatmasker.org

^4^https://github.com/nextgenusfs/funannotate, accessed April 14, 2024

^5^ https://megasun.bch.umontreal.ca/cgi-bin/RNAweasel/

^6^ http://genomes.cicbiogune.es/SECRETOOL

^7^ www.phi-base.org
